# Developing a Machine Learning Model for Personalized, Predictor-Centric, Adaptive Intervention for Vaping Cessation in Young People: Secondary Data Analysis of Smartphone App Data

**DOI:** 10.3390/ijerph23040527

**Published:** 2026-04-18

**Authors:** Anasua Kundu, Peter Selby, Daniel Felsky, Theo J. Moraes, Lynn Planinac, Michael Chaiton

**Affiliations:** 1 Institute of Medical Science, University of Toronto, Toronto, ON M5S 1A8, Canada; 2 Centre for Addiction and Mental Health, Toronto, ON M6J 1H4, Canada; 3 Dalla Lana School of Public Health, University of Toronto, Toronto, ON M5T 3M7, Canada; 4 Department of Family and Community Medicine, University of Toronto, Toronto, ON M5G 1V7, Canada; 5 Department of Psychiatry, University of Toronto, Toronto, ON M5T 1R8, Canada; 6 The Hospital for Sick Children (SickKids), Toronto, ON M5G 1E8, Canada; 7 Department of Paediatrics, University of Toronto, Toronto, ON M5G 1X8, Canada; 8 Institute of Health Policy, Management and Evaluation, University of Toronto, Toronto, ON M5T 3M6, Canada

**Keywords:** e-cigarettes, vaping cessation, machine learning, prediction, behavior therapy

## Abstract

**Highlights:**

**Public health relevance—How does this work relate to a public health issue?**

- Despite an increasing number of young people trying to quit e-cigarettes, interventions for vaping cessation are still lacking.
- Incorporating barriers of successful cessation to develop predictor-centric personalized behavioral interventions may improve the efficacy of vaping cessation interventions.

**Public health significance—Why is this work of significance to public health?**

- We built a machine learning model with only five features: self-confidence in quitting, intention to quit, average e-liquid used per week, time to first vape and mood trend during challenge.
- The model can predict individual probability of short-term vaping relapse and identify the person-specific barriers to successful cessation.

**Public health implications—What are the key implications or messages for practitioners, policy makers and/or researchers in public health?**

- We presented predictive risk-score based stratification, which can inform the development of tailored quit plans for vaping cessation among young people.
- The model can guide the development of personalized, predictor-centric, adaptive behavioral interventions to minimize barriers to successful cessation.

**Abstract:**

Although increasing numbers of young people are trying to quit e-cigarettes, personalized tools to support vaping cessation remain limited. We aimed to build a machine learning model to predict individual probability of short-term relapses and identify person-specific barriers to successful cessation. Data were taken from the “Stop Vaping Challenge” smartphone app. We included past 30-day e-cigarette users aged 15–35 years (*n* = 311) who completed 387 quit challenges. Feature selection minimized number of predictors while maximizing predictive ability. We built multiple GBM survival models with different sets of predictors to predict time to vaping relapse. The five-feature model yielded the best performance (C-index 0.751), thereby was selected as the final model. These five features were: self-confidence in quitting, intention to quit, average e-liquid used per week, time to first vape and mood trend during challenge. We stratified the challenges by the individual relapse risk by 7 days into low-, medium-, and high probability of quit success. This approach can inform tailored quit plans for vaping cessation. SHAP analysis demonstrated individual-level barriers to cessation, which can guide the development of personalized, predictor-centric, adaptive behavioral interventions. However, future research is needed to implement the model in real-world settings and evaluate its effectiveness and generalizability.

## 1. Introduction

Amid high and rising nicotine e-cigarette use among young people [1], a growing number are also attempting to quit vaping. About 41% of Canadian and 45% of the United States (US) youth e-cigarette users reported at least one quit attempt in the year 2023 [2]. However, a recent meta-analysis of seven randomized controlled trials (RCTs) found that people who received any intervention were approximately 50% more likely remain abstinent for 7 days compared to those receiving no intervention [3].

Digital health interventions like computer-based programs, smartphone apps, and internet-based interventions are a form of healthcare delivery that are particularly appealing, cost-effective and acceptable among young people [4]. Two-thirds of American adolescents and young adults reported using health-related mobile health (mHealth) apps [5]. Recent systematic reviews and meta-analysis showed that mHealth apps significantly improve mental health outcomes, especially when combined with traditional care [6,7]. Furthermore, with the growing use of artificial intelligence (AI) in medicine, machine learning (ML)-based mHealth apps, particularly those targeting behavioral adjustments and treatment approaches, are a big step forward to implement personalized healthcare [8,9].

Several ML-based real-life digital health interventions have demonstrated effectiveness in predicting and managing depression, psychological conditions, lifestyle change, and smoking cessation [10]. Notably, in the context of smoking cessation, ML-informed approaches have consistently shown strong performance in predicting treatment outcomes, supporting its potential for delivering personalized care [11,12,13]. Meta-analysis findings indicate that personalized or interactive interventions improved the likelihood of abstinence in both short- and long-term smoking cessation [14,15]. Personalization may enhance intervention effectiveness by tailoring support to an individual’s capabilities, opportunities, and motivation [16], and thereby increasing engagement and the likelihood of successful cessation [15]. Randomized controlled trial testing an AI-powered real-time, adaptive, personalized intervention as a smoking cessation app, “Quit Sense”, showed 4.57 times higher odds of abstinence at 6 months among the app users compared to participants receiving usual care [17]. Unfortunately, no such intervention exists for vaping cessation [3]. All four digital interventions identified in a recent systematic review were designed to provide motivational text counseling or smartphone app-based telehealth services [3]. None of these studies focused on predictors of vaping cessation and used predictor-centric personalized behavioral modifications to improve treatment outcome. Identifying barriers of cessation and delivering personalized behavioral modifications to address these barriers have proven effective for smoking cessation [18] and warrant investigation in the context of vaping cessation.

In our previous study, we used the longitudinal data from an existing vaping cessation smartphone app and conducted an exploratory predictive analysis to determine the most important predictors of vaping relapse [19]. This work extends our previous research, where we aimed to build a ML-based model suitable for digital interventions that can predict each user’s probability of short-term vaping cessation and identify person-specific barriers before participating in a quit attempt. In addition, the model’s ability to identify the person-specific barriers were used to highlight its potential to inform the development of personalized, predictor-centric, adaptive interventions or tailored quit plans for each user.

## 2. Materials and Methods

### 2.1. Data Source

Data for this study were collected from a vaping cessation smartphone app named “Stop Vaping Challenge”. The app was launched by the Ontario Tobacco Research Unit, University of Toronto in June 2021 and is available in both Apple and Android app stores [20]. It allows users to take part in ‘quit challenges’ for as long as they wish, with the help of a timer. Upon sign-up for the app, users are requested to complete a baseline survey. During a quit challenge, they are requested to enter ratings of their mood and cravings hourly for the first 3 h and then daily until the challenge ends. An individual user can take part in multiple quit challenges. For each user, a unique identifier was used to link baseline survey data to mood and craving responses for quit challenges. In this analysis, we included users aged 15–35 years who reported using e-cigarettes at least once in the past 30 days at baseline. Data were collected from 11 November 2021 up to 29 April 2025. In order to maintain high-quality data and to distinguish between attrition and continued abstinence [21], we included data of those challenges that lasted at least 5 min (to avoid non-starters) and elicited data on self-reported mood and craving ratings. The analytic sample size was 311 participants, who together attempted 387 independent quit challenges. Ethical permission for this study was approved by the University of Toronto Research Ethics Board and all participants provided written informed consent before participating in this study. The analysis plan for identifying the initial predictors was preregistered in the Open Science Framework (OSF) [22]. The statistical analyses were mainly performed using R version 4.3.0. Building the final model was done using Python version 3.13.7. Relevant Python codes and the deidentified dataset needed to build the final model are available on the GitHub repository (https://github.com/anasuakundu/Stop-Vaping-Challenge-final-ML-model (accessed on 3 April 2026)).

### 2.2. Candidate Predictors and Outcome Measures

A total of 15 features, 13 categorical and 2 continuous, were included as potentially predictive variables. These features were selected based on our findings from the exploratory predictive analysis conducted on the same dataset [19]. In this previous analysis, initially we included 37 features representing a range of variables representing various individual and environmental factors [19] (see Table S1). We compared the performances of multiple ML algorithms to predict time to vaping relapse based on these 37 features. The Gradient boosting machine (GBM) survival algorithm achieved the best performance, and the model identified 15 features as the top-most important predictors [19]. These 15 predictors were self-confidence in quitting, intention to quit, monthly vaping expense, pod depletion time, time to first vape, past 30-day alcohol drinking, past-month frequency of vaping, initial craving during challenge, initial mood during challenge, craving trend during challenge, mood trend during challenge, other reasons for quitting (reasons excluding cost, health or addiction concerns, or pressure from family or peer pressure), average e-liquid used per week, self-perceived addiction, and sexual orientation. Of these, four features were extracted from the mood and craving ratings reported during the challenges, while 11 features were from the baseline survey. Details of these 15 features are presented as Table S2. All included predictors had sufficient variability, with all percentages of unique values > 5% for the categorical variables [23].

The primary study outcome was time to vaping relapse, defined as the duration of a single quit challenge. Each challenge was assigned a binary status indicator, ‘relapsed’ or ‘still abstinent’ challenges. Since app users were instructed to physically stop a challenge if they resumed vaping, a terminated challenge was deemed to indicate relapse. ‘Relapse’ status was further confirmed by asking users whether they had vaped. For challenges that were still active at the time of data extraction, we labeled those challenges as ‘still abstinent’ but censored the follow-up time at the last recorded mood or craving entry, confirming abstinence up to that point.

### 2.3. Data Pre-Processing

For the purpose of this study, each quit challenge was treated as a separate observation. The distribution of number of quit challenges among participants was relatively balanced. Most quit challenges (*n* = 259, 67%) were initiated by unique individuals, with a maximum of six challenges completed by a single individual. There was a total of 5.8% missing data among all predictors, with four having >5% missing observations. After confirming the assumption that missing at random was plausible, we imputed missing data using logistic regression and predictive mean matching for categorical and continuous variables respectively. Continuous variables were standardized prior to model fitting.

### 2.4. Feature Selection

We intended to get an optimal set of predictors that in addition to being minimal in number also yield a reasonable performance when incorporated in a ML model. To achieve that, we applied combinations of two different feature selection methods- minimum redundancy maximal relevance (mRMR), and random survival forest (RSF). mRMR filters features by selecting variables with high mutual information with the outcome while penalizing redundancy among selected features, resulting in a compact, most relevant to the outcome, and non-redundant feature set [24]. RSF performs implicit feature selection through random feature subsampling at each node and survival-based splitting criteria. Predictors that better separate survival outcomes are selected more frequently across trees and contribute more to ensemble predictions [25]. Previous studies have demonstrated the utility of both methods for feature selection, particularly when combinations of different feature selection approaches are applied [26,27,28]. Initially, 8 features out of the 15 features were selected by applying mRMR, while RSF ranked these 8 features according to their feature importance. Finally, to compare performance, we built multiple ML models first with the initial 15 features, then with the 8 features achieved post-mRMR, and with progressively smaller subsets of top-ranked predictors (i.e., 6, 5, and 4 features) identified using RSF, retaining predictors until no further improvement in model performance was observed.

### 2.5. Machine Learning Models

We used the GBM survival [29] algorithm to build the ML models because this algorithm yielded the best performance in our initial analysis [19]. GBM survival creates an ensemble of decision tree models, where decisions from multiple models are aggregated rather than considered in isolation, significantly improving overall predictive capability. To handle right-censored data, we used the ‘gbm’ framework with Cox partial likelihood (distribution = ‘coxph’) as the loss function [30,31]. To mitigate overfitting and permit unbiased hyperparameter optimization, 10 × 5-fold nested cross-validation (nCV) was used [32]. While traditional k-fold cross-validation holds-out a fixed test dataset for performance estimation, the nCV approach maximizes the use of the entire dataset by iteratively cycling through different combinations of training and testing data in a sequence of both outer and inner subset loops [32]. This also minimizes within-sample validation error, guards against overfitting, and improves generalizability.

The entire dataset of quit challenges (*n* = 387) was first randomly split into training (*n* = 348, 90%) and test (*n* = 39, 10%) sets across 10 outer folds. Classes were reasonably balanced across folds, as the relapse outcome was common (72% prevalence of relapses events). With each outer data split, the data were again randomly split into training (*n* = 279, 80%) and validation (*n* = 69, 20%) sets, constituting the five inner folds. A grid search approach [33] was used to optimize model hyperparameters within inner folds, and optimal selections were carried forward to fit and test each model in the outer folds. Model performance was evaluated on outer test sets using Harrell’s concordance index (C-index) [34]. The C-index provides an overall assessment of a model’s discriminative ability: values > 0.7 are generally considered good, 0.6–0.7 moderate, and <0.6 poor [35]. We calculated the average C-index across the 10 outer folds and also unidentified the model out of the 10 outer fold models that yielded the highest C-index. Finally, the feature set that built the model with the highest C-index was chosen as the final model. The outer fold train and test dataset used for building this final model was preserved for further analysis.

### 2.6. Final Model and Interpretation

Python is widely used for software development due to its flexibility as a multi-purpose programming language, strong developer community support, and extensive use in ML applications, whereas R is more commonly used for data analytics and visualization [36,37,38]. Therefore, to facilitate broader implementation of the ML model across digital interventions, we built the final GBM survival model in the Python interface using the preserved train and test dataset, same hyperparameters, and applying the ‘GradientBoostingSurvivalAnalysis’ framework [39]. Model performance was measured with a risk-score-based Harrell’s C-index and individual probability of relapse by 7 days for each quit challenge. For interpretation of the so-called ‘black box’ ML model [40], we used Shapley Additive ExPlanations (SHAP) method. SHAP values are a recent application of the decades-old game-theoretic concept of Shapley values, which quantify the average marginal contribution of each feature to a model’s prediction [40]. We used a model-agnostic SHAP approach [41] to calculate SHAP values from the withheld outer fold test dataset of the final model. A positive SHAP value for a given feature and individual indicates a positive contribution to the probability of relapse, while a negative value indicates a negative contribution. Using the average absolute SHAP values across all participants for a given feature, that feature’s overall contribution to the model prediction can be summarized. SHAP dependence plots [42] visualized these variability and non-linearity of effects of the final predictors. Finally, this approach allowed us to demonstrate the power of ML paired with SHAP analysis by generating SHAP waterfall plot, which illustrates the magnitude and direction of each predictor’s contribution for a single participant, aiding in identifying barriers of successful cessation.

### 2.7. Sensitivity Analysis

To assess the influence of within-person dependence [43], we restricted the dataset to the first quit attempt per individual (*n* = 311) and included only the final 5 features. The GBM survival model was built on this restricted dataset, and we compared the performance with the final 5-feature model.

## 3. Results

### 3.1. Sample Characteristics

Of 311 participants, the majority were identified as women (*n* = 174, 55.9%), heterosexual (*n* = 228, 73.3%), and white (*n* = 194, 66.9%) with an average age of 22.62 ± 5.07 years. More than half of the participants lived in countries other than Canada and the US. The average number of quit attempts was 1.24 ± 0.66 per person. While the overall duration (median) of abstinence was <1 day. Out of the 387 quit challenges, 71.8% (*n* = 278) relapsed (Table 1). The average duration of abstinence was 5.85 ± 20.07 days in the still abstinent challenges and 3.33 ± 13.58 days in the relapsed challenges. The highest duration of abstinence recorded were 171 (censored time-point) and 194 days in the still abstinent and relapsed challenges respectively. Although the difference was not statistically significant, about 12.8% (*n* = 14) of the abstinent challenges lasted for at least 7 days, while only 9.71% (*n* = 27) of the relapsed challenges lasted the same. Relapsed quit challenges had significantly lower self-confidence in quitting (*p* < 0.001), lower intention to quit within next month (*p* = 0.008), lower initial cravings during challenges (*p* = 0.017), but higher rate of reporting other reasons to quit (*p* = 0.002) compared to still abstinent quit challenges. Relapsed challenges also showed higher rates of reporting increasing cravings (*p* = 0.058) and lower rates of mood improvement (*p* = 0.139) over time. Additionally, inconsistent mood and craving trends were more frequently observed in relapsed challenges compared to still abstinent challenges (Table 1).

### 3.2. Feature Selection Findings

mRMR retained the following 8 features out of the 15 features: self-confidence in quitting, intention to quit, time to first vape, past 30-day alcohol drinking, initial craving during challenge, mood trend during challenge, other reasons for quitting, and average e-liquid used per week (Table S3). On RSF, the importance scores of past 30-day alcohol drinking and initial craving during challenge were found close to zero hence deemed the least important. RSF ranked the remaining six features in the following order (In descending order of importance): intention to quit, self-confidence in quitting, mood trend during challenge, average e-liquid used per week, time to first vape, and other reasons for quitting (Table S3).

### 3.3. Model Performances and Sensitivity Analysis Findings

We built five GBM survival models with the initial 15 features, then 8 features (post-mRMR), 6 features (post-mRMR-RSF), 5 features (post-mRMR-RSF), and 4 features (post-mRMR-RSF) respectively. Of them, the six-feature and five-feature models had the highest average C-indices across the 10 outer folds (C-index 0.658 ± 0.059 and 0.654 ± 0.067 respectively) (Table 2 and Figure S1). The average C-index for the 4-feature model (0.638 ± 0.069) was lower compared to that of the five-feature model, hence we stopped building models with fewer features. Of all models built in the outer folds, the best performance was seen with the five-feature model (C-index 0.774) (Table 2). Therefore, we chose this one as the final model. However, the C-index of this model in Python interface was 0.751. This difference in C-index between Python and R implementation is expected because the Python model directly optimized survival-specific loss function, whereas the R implementation boosted a Cox proportional hazard model. Consequently, even with similar tuning parameters, the underlying loss functions and algorithmic details differed, leading to variations in model performance metrics or Harrell’s C-index, as shown in a previous study [44]. The computational time for building the model and applying SHAP with Python was 4 s.

In a sensitivity analysis, the GBM survival model built on the restricted dataset (*n* = 311) demonstrated lower performance relative to the final five-feature model. The average C-index was 0.608 ± 0.048, respectively, while the highest C-index achieved was 0.673 (Table S4).

### 3.4. Interpretation of the Final ML Model

**Figure 1:**
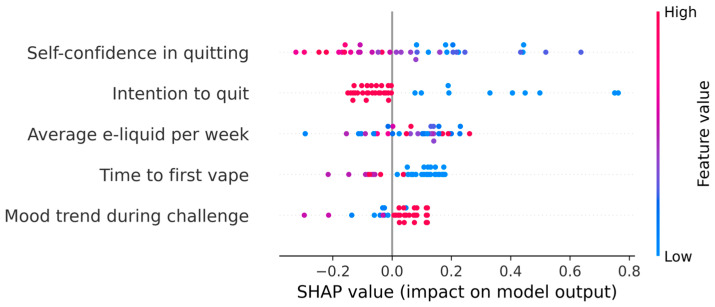
SHAP summary plot for the final five features predicting time to vaping relapse.

The final ML model was built with the following five features: self-confidence in quitting, intention to quit, average e-liquid used per week, time to first vape, and mood trend during challenge (Figure 1).

**Figure 2:**
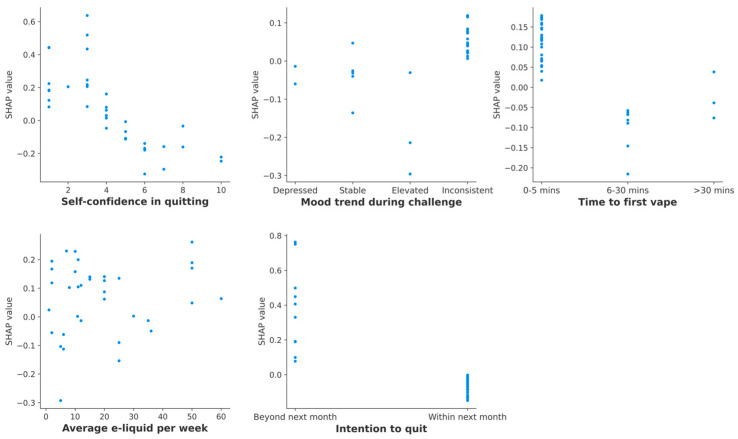
SHAP dependence plot showing effects on SHAP values by the final five features (each blue dot represents the SHAP value for an individual challenge).

Self-confidence in quitting had the highest average SHAP value indicating highest impact on the model output. The risk of relapse decreased with higher self-confidence in quitting, particularly when rated as ≥6 on a scale of 0–10 (Figure 2). Intention to quit was the second most important predictor, whereas those who intended to quit within next month showed lower risk of relapse compared to those who intended to quit in the future, but not within the next month. A non-linear association was observed between e-liquid consumption and relapse risk. Using e-liquid on an average <20 mL/week was associated with both increased and decreased risk, 20–40 mL/week with reduced risk, and >40 mL/week with increased risk of relapse. In case of time to first vape, those who vaped within 5 min of waking up showed higher risk of relapse, while having first vape within 6–30 min of waking up was associated with lower risk of relapse. However, having first vape after 30 min of waking up showed neither higher nor lower risk of relapse. People whose mood became increasingly better during the challenges had a lower risk of relapse, while those with an inconsistent mood trend had a higher risk. Stable mood throughout the challenges was not associated with relapse risk (Figure 2).

**Figure 3:**
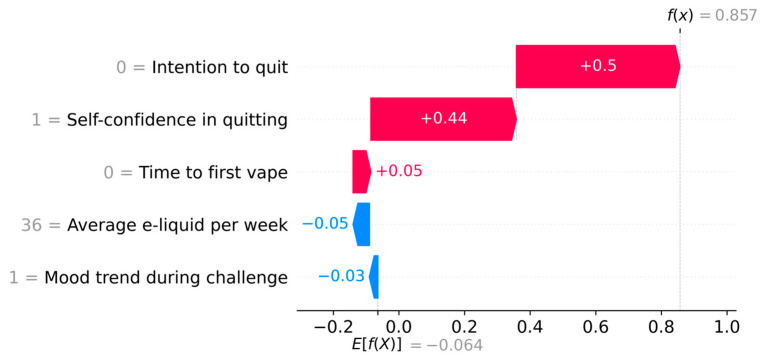
SHAP waterfall plot showing combined event risk of vaping relapse for a single quit challenge (the red-colored predictors increased risk of relapse, while the blue-colored predictors decreased the risk of relapse).

We categorized all challenges according to the probability or risk of relapse by 7 days, whereas ≤25% risk was defined as ‘high probability of quit success’, 26–74% risk as ‘medium probability of quit success, and ≥75% risk as ‘low probability of quit success’. Figure 3 presents how the model can predict the individual probability of relapse for a single representative quit challenge by a single individual. Based on the information gathered on the five features, the probability of relapse by 7 days was 98.7% for this challenge, which puts it in the ‘low probability of quit success’. The barriers to successful cessation were no intention to quit within next month, low self-confidence in quitting, and time to first vape within 0–5 min. On the other hand, using 36 mL/week e-liquid on average, and stable mood trend during the challenge acted as facilitators of cessation.

## 4. Discussion

We built a ML model of good performance with only five predictors and Python codes which can be incorporated in any digital intervention (i.e., smartphone app or web-based intervention) targeting personalized vaping cessation in young people aged 15–35 years. In addition to predicting individual probability of short-term relapse (by 7 days), the model identifies barriers of successful cessation that are driving the person to relapse. The limited number of predictors and low computational burden support the feasibility of implementing the model in digital interventions. We uploaded the Python codes, the deidentified train and test dataset, and the questionnaire that are needed to build the model in the GitHub (https://github.com/anasuakundu/Stop-Vaping-Challenge-final-ML-model (accessed on 3 April 2026)) open access platform.

Self-confidence in quitting and intention to quit appeared as the two top-most important predictors of vaping relapse. Self-confidence or self-efficacy as a predictor of health behavior is not a new concept. In Bandura’s social cognitive theory, self-efficacy influences an individual’s decision to engage in a specific behavior, such as refraining from e-cigarette use, and their effort and persistence in the face of challenges [45]. Previous research showed that use of mHealth apps positively impacted self-efficacy and intention to quit among smokers who made a quit attempt [46], which eventually improved treatment outcome [47,48]. Hence, self-efficacy and intention to quit are considered integral components of personalized interventions [15]. A Cochrane meta-analysis showed that incorporating a psychosocial mood management component in smoking cessation intervention improves likelihood of cessation [49], which is also supported by our finding in respect of vaping cessation. Other predictors, such as average e-liquid used per week and time to first vape, overall reflect the well-established association between higher nicotine dependence and greater difficulty in quitting [50]. The conflicting findings in the <20 mL/week average e-liquid users and >30 min time to first vape group are not surprising, since smoking cessation research showed similar patterns among intermittent smokers, who typically consume fewer cigarettes and delay their first daily cigarette, yet still experience difficulty quitting [51]. Gradual reduction in use, rather than abrupt cessation, is an approved and effective smoking cessation strategy [52], supporting its application to personalized vaping cessation interventions. However, rather than focusing on a single predictor, the ML model evaluates the individual effects of all predictors, accounts for complex non-linear interactions among them, and combines these effects to estimate each user’s probability of short-term (7-day) relapse.

An important aspect of this study is that we prioritized not only the predictive power but also selection of a minimal set of features for building the model. Balancing this trade-off ensures the model’s suitability, feasibility, and utility for real-life implementation, which is a recommended approach for building clinical prediction models [53]. To our knowledge, this model is the first to support personalized, predictor-centric, adaptive behavioral interventions for vaping cessation among young people. Once incorporated in a digital intervention, the model will be built with the training dataset, and any new user data will constitute the test dataset. The new user will take part in an initial quit challenge, where they will record their mood hourly for first 3 h and then daily for the remainder of the challenge. These data will be used to compute one of the five predictors, mood trend, during the challenge. In addition, they will also complete a small four-item pre-quit survey (see Table S5) and provide information on the remaining four predictors: self-confidence in quitting, intention to quit, average e-liquid used per week, and time to first vape. Data on these five predictors will constitute the test data for the new user, and the model will predict probability of relapse by 7 days and person-specific barriers of cessation for this new user, as shown in Figure 3. Based on these findings, tailored suggestions targeting behavior change to address the identified barriers could be provided to the individual prior to a quit attempt, thereby increasing the probability of successful cessation. If the person again fails to quit, the entire process can be repeated, updating the prediction with new data collected through the mood recordings in the preceding challenge and the updated pre-quit survey, thereby adapting the behavioral intervention to the specific quit challenge. This type of personalized, adaptive intervention delivered via a smartphone app has been evaluated through a randomized controlled trial and was found to be 1.81 times more effective in achieving smoking cessation compared with a standard smartphone app [18].

The predicted individual probability of relapse was also used to categorize the challenges into three categories—low-, medium-, and high probability of quit success. This type of ML-based risk stratification to prevent harmful events has previously been tested for other health conditions [54,55]. Moreover, stratifying e-cigarette users by relapse risk can help inform the delivery of triaged or targeted interventions. For example, people with low and medium probability of quit success can be offered a more intensive quit plan, such as gradually tapering off e-cigarette use to complete cessation, behavioral therapy, counseling, seeking expert help and medications including nicotine replacement therapy. On the other hand, people with a high probability of quit success might be suggested to go ‘cold turkey’ and seek further help if needed. In fact, this triaged quit plan based on the ML model is currently under development to be incorporated in the ‘Stop Vaping Challenge’ app [56]. Targeted intervention approaches have previously been applied for smoking cessation [57,58] and showed promises in achieving continuous smoking abstinence in a cluster randomized controlled trial [58].

It should be noted that although the performance of the model was good (C-index 0.751), it has limitations as a clinical tool. First of all, the model has not yet been tested by a randomized controlled trial, hence we cannot comment on the effectiveness of the model as an intervention rather than a predictive model. Moreover, the small sample size is a barrier to the generalizability of the model, although we applied nCV technique to maximize the use of the dataset. The outcome is based on self-reported data, therefore the abstinence status was not objectively verified. Furthermore, we censored the duration of the ‘abstinent’ challenges at the last mood or craving entry. However, some participants may have disengaged from the app and subsequently relapsed without reporting it. These limitations can be addressed by developing our model in a larger and more comprehensive dataset and testing it through a randomized controlled trial using objective outcome measures. External validation of a ML model on an independent sample is essential for reproducibility across populations [59], which was absent in our model. As the model itself is available for universal access, we encourage fellow researchers to test it on an external validation set. Finally, in both previous and current analysis, we only measured mood and cravings during an ongoing quit challenge. However, other potential triggers of relapse may also be involved, such as exposure to peer and family vaping, seeing other people vaping, and exposure to e-cigarette advertisements and marketing during quit attempts. These factors should be considered for inclusion in future research. There are additional avenues for future research. For example, the utility of this model across subpopulations defined by age, sex, race, income, and probability of quit success warrants further investigation. The model predicts only short-term relapse, reflecting the relatively brief duration of most challenges, and therefore cannot determine whether the identified predictors also influence long-term cessation success. Furthermore, as a predictive modeling study, it does not establish causal relationships between the predictors and outcomes, which should be further investigated by future longitudinal or experimental studies.

## 5. Conclusions

Our ML model represents an important first step toward ML-based digital intervention targeting vaping cessation among young people. In addition to providing individual probability of short-term relapse, the model also can serve as a tool for development of personalized, predictor-centric, adaptive intervention aimed at minimizing barriers to cessation and designing risk-stratification-based quit plans to facilitate successful quitting. Future work should focus on evaluating the effectiveness of this approach in real-world settings and further strengthening the model to enhance its generalizability.

## Figures and Tables

**Table 1:** Summary statistics of quit challenges stratified by quitting status (*n* = 387).

Characteristics	Still Abstinent (*n* = 109)	Relapsed (*n* = 278)	*p*-Value
**Past-month frequency of vaping at baseline (days), mean (SD)**	26.94 (7.29)	27.00 (6.76)	0.952
**Time to first vape after waking up, *n* (%)**			0.298
Less than 5 min	57 (52.3)	160 (57.6)	
6–30 min	38 (34.9)	75 (27.0)	
>30 min	14 (12.8)	43 (15.5)	
**Self-perceived addiction, *n* (%)**			0.512
Less addicted	30 (27.8)	66 (23.9)	
Very addicted	78 (72.2)	210 (76.1)	
**Intention to quit at baseline, *n* (%)**			0.008
Beyond next month	12 (11.1)	66 (23.8)	
Within next month	96 (88.9)	211 (76.2)	
**Self-confidence in quitting, mean (SD)**	6.13 (2.78)	4.93 (2.72)	<0.001
**Monthly vaping expense (dollars), mean (SD)**	52.82 (40.40)	50.58 (35.55)	0.612
**Average e-liquid vaped per week (ml), mean (SD)**	19.02 (14.86)	16.77 (14.97)	0.282
**Pod depletion time (days), mean (SD)**	4.65 (3.88)	4.57 (4.28)	0.874
**Past 30-day alcohol drinking at baseline, *n* (%)**	80 (73.4)	175 (62.9)	0.067
**Other reasons for quitting (excludes cost, health, addiction concerns, and peer or family pressure), *n* (%)**	74 (67.9)	231 (83.1)	0.002
**Initial mood during challenge, mean (SD)**	5.40 (2.51)	5.32 (2.30)	0.744
**Initial craving during challenge, mean (SD)**	6.14 (2.89)	5.33 (3.01)	0.017
**Mood trend during challenge, *n* (%)**			0.139
Depressed	20 (18.3)	34 (12.2)	
Stable	15 (13.8)	39 (14.0)	
Elevated	12 (11.0)	18 (6.5)	
Inconsistent	62 (56.9)	187 (67.3)	
**Craving trend during challenge, *n* (%)**			0.058
Decreased	11 (10.1)	12 (4.3)	
Stable	21 (19.3)	38 (13.7)	
Increased	13 (11.9)	44 (15.8)	
Inconsistent	64 (58.7)	184 (66.2)	

Note: Feature values presented in this table are based on the unimputed dataset. Abbreviation: SD, standard deviation.

**Table 2:** Performance of the GBM survival models built across 10 outer folds predicting time to vaping relapse (*n* = 387).

Model	Average Harrell’s C-Index (SD)	Best Harrell’s C-Index
15-feature model	0.636 (0.056)	0.716
8-feature model (post-mRMR)	0.663 (0.065)	0.736
6-feature model (post-mRMR-RSF)	0.658 (0.059)	0.758
5-feature model (post-mRMR-RSF)	0.654 (0.067)	0.774
4-feature model (post-mRMR-RSF)	0.638 (0.069)	0.758

Abbreviation: C-index, Concordance index; GBM, gradient boosting machine; mRMR, minimum redundancy maximal relevance; RSF, random survival forest; SD, standard deviation.

## Data Availability

The deidentified datasets to build the final machine learning model is publicly available at GitHub (https://github.com/anasuakundu/Stop-Vaping-Challenge-final-ML-model (accessed on 3 April 2026)). The original raw dataset is available in University of Toronto Dataverse and can be accessed upon further request to the e-mail (anasu.kundu@mail.utoronto.ca).

## Supplementary Materials

The following supporting information can be downloaded at: https://www.mdpi.com/article/10.3390/ijerph23040527/s1, Table S1: Variables (*n* = 37) included in previous exploratory predictive analysis [19]; Table S2: Details of the predictor variables included in the initial analysis (*n* = 15); Table S3: Predictors selected in each model; Table S4: Sensitivity analysis findings: Performance of the model built with final 5-features and model built on the restricted dataset (including only first quit attempt per individual); Table S5: Pre-quit survey questionnaire; Figure S1. Harrell’s C-index across 10 outer fold test sets for all GBM survival models.

## Abbreviations

The following abbreviations are used in this manuscript:

GBM	Gradient Boosting Machine
ML	Machine learning
mRMR	Minimum redundancy maximal relevance
nCV	Nested cross-validation
RSF	Random Survival Forest
SHAP	Shapley Additive ExPlanations
