# Supplementary material for: Developing a Machine Learning Model for Personalized, Predictor-Centric, Adaptive Intervention for Vaping Cessation in Young People: Secondary Data Analysis of Smartphone App Data
Source: Int J Environ Res Public Health. 2026 Apr 18;23(4):527. doi: 10.3390/ijerph23040527 (PMC13116711; doi:10.3390/ijerph23040527)

Supplementary Materials

Developing a machine learning model for personalized, predictor-centric, adaptive intervention for vaping cessation in young people: secondary data analysis of smartphone app data.

Contents:

Table S1. Variables (*n* = 37) included in previous exploratory predictive analysis [19].

Table S2. Details of the predictor variables included in the initial analysis (*n* = 15).

Table S3. Predictors selected in each model

Table S4. Sensitivity analysis findings: Performance of the model built with final 5-features and model built on the restricted dataset (including only first quit attempt per individual).

Table S5. Pre-quit survey questionnaire.

Figure S1. Harrell's C-index across 10 outer fold test sets for all GBM survival models

Table S1. Variables (*n* = 37) included in previous exploratory predictive analysis [19].

Socio-demographic factors	Measures of e-cigarette use	Measures of dependence	Vape product characteristics	Quit-related factors	Other substance use patterns	Social activity levels	Psychological and health	Mood and cravings during challenges
Age	Past month frequency of vaping	Time to first vape	Device type	Reasons for quitting-addiction concern	Past 30-day cigarette smoking	Peer vaping	Believing vaping is safer than smoking	Initial mood during challenge
Gender	Puffs per session	Self-perceived addiction	Flavor used	Reasons for quitting-cost	Past 30-day cannabis use		Perceived general health	Initial craving during challenge
Sexual orientation	Monthly vaping expense	Overall cravings at baseline	Nicotine strength	Reasons for quitting-health concern	Past 30-day alcohol drinking		Perceived mental health	Mood trend during challenge
Race	Average e-liquid per week			Reasons for quitting-family or peers demand	Past 30-day other tobacco products use		Overall mood at baseline	Craving trend during challenge
	Pod depletion time			Reasons for quitting-others			Perceived stress	
				Intention to quit				
				Past year quit attempts				
				Self-confidence in quitting				

Table S2. Details of the predictor variables included in the initial analysis (*n* = 15).

Variable	Description	Levels/characteristics	Data source
**Socio-demographic factors**			
Sexual orientation	Sexual orientation	0= Heterosexual, 1= LGBTQ, 2= asexual or undisclosed	Baseline survey
**Measures of e-cigarette use**			
Past month frequency of vaping	Frequency of current vaping in past 30 days	Numeric, days in past 30 days	Baseline survey
Monthly vaping expense	Past month spending on vaping	Numeric, dollars	Baseline survey
Average e-liquid per week	Average e-liquid vaped per week	Numeric, ml	Baseline survey
Pod depletion time	On average, how long a single vape pod lasts	Numeric, days	Baseline survey
**Measures of dependence**			
Time to first vape	Time to first vape after waking up	0= 0-5 mins, 1=6-30 mins, 2= >30 mins	Baseline survey
Self-perceived addiction	Self-perceived addiction to e-cigarette	0= Less addicted, 1= Very addicted	Baseline survey
**Quit-related factors**			
Reasons for quitting-others	Reason for quitting- others (including lost interest, did not help to quit smoking, it was hard, to test myself, to take this challenge with friends, challenging myself, others; but excluding cost, health or addiction concerns, or pressure from family or peer pressure)	0=no, 1=yes	Baseline survey
Intention to quit	Intention to quit at baseline	0= Beyond next month (within next 6 months or beyond 6 months), 1= Within next month,	Baseline survey
Self-confidence in quitting	Confidence in quitting vaping within next month on a scale of 1-10	Numeric, higher score indicates higher confidence.	Baseline survey
**Other substance use patterns**			
Past 30-day alcohol drinking	Drink alcohol in past 30 days	0=no, 1=yes	Baseline survey
**Mood and cravings during challenges**			
Initial mood during challenge	First mood recording during a single challenge	Numeric, range 0-10	Mood and cravings entry during challenge
Initial craving during challenge	First craving recording during a single challenge	Numeric, range 0-10	Mood and cravings entry during challenge

Mood trend during challenge	Transition of mood during a single challenge, difference between first and last recordings	0= depressed, 1= stable or no change, 2= elevated, 3= inconsistent	Mood and cravings entry during challenge
Craving trend during challenge	Transition of craving during a single challenge, difference between first and last recordings	0= decreased, 1= stable or no change, 2= increased, 3= inconsistent	Mood and cravings entry during challenge

Table S3. Predictors selected in each model.

	15-feature model	8-feature model (post-mRMR)	6-feature model (post-mRMR-RSF)	5-feature model (post-mRMR-RSF)	4-feature model (post-mRMR-RSF)
Self-confidence in quitting	✓	✓	✓	✓	✓
Intention to quit	✓	✓	✓	✓	✓
Monthly vaping expense	✓				
Pod depletion time	✓				
Time to first vape	✓	✓	✓	✓	
Past 30-day alcohol drinking	✓	✓			
Past month frequency of vaping	✓				
Initial craving during challenge	✓	✓			
Initial mood during challenge	✓				
Craving trend during challenge	✓				
Mood trend during challenge	✓	✓	✓	✓	✓
Reasons for quitting-others	✓	✓	✓		
Average e-liquid per week	✓	✓	✓	✓	✓
Self-perceived addiction	✓				
Sexual orientation	✓				

Table S4. Sensitivity analysis findings: Performance of the model built with final 5-features and model built on the restricted dataset (including only first quit attempt per individual).

Model	Sample size	Average Harrell's C-index (sd)	Best Harrell's C-index
**5-feature model**	387	**0.654 (0.067)**	**0.774**
Restricted dataset model	311	0.608 (0.048)	0.673

Abbreviation: C-index, Concordance index

Table S5. Pre-quit survey questionnaire.

Features	Questions	Values	Type of feature
Self-confidence in quitting	**How confident are you that you can quit vaping e-cigarettes within the next month and stay quit for good?**	1 Not at all confident<br>2<br>3<br>4<br>5<br>6<br>7<br>8<br>9<br>10 100% confident	Continuous
Intention to quit	**Are you planning to quit vaping completely?**	0- Beyond next month but sometime in the future<br>1- Within next month	Categorical
Average e-liquid per week	**On average, how much e-liquid do you vape each week?**	Enter number of ml only (enter a number between 0 and 65)	Continuous
Time to first vape	**On days that you can vape freely, how soon after you wake up do you have the first vape of the day**	0- 0-5 minutes<br>1- 6-30 minutes<br>2- >30 minutes	

Figure S1. Harrell's C-index across 10 outer fold test sets for all GBM survival models.

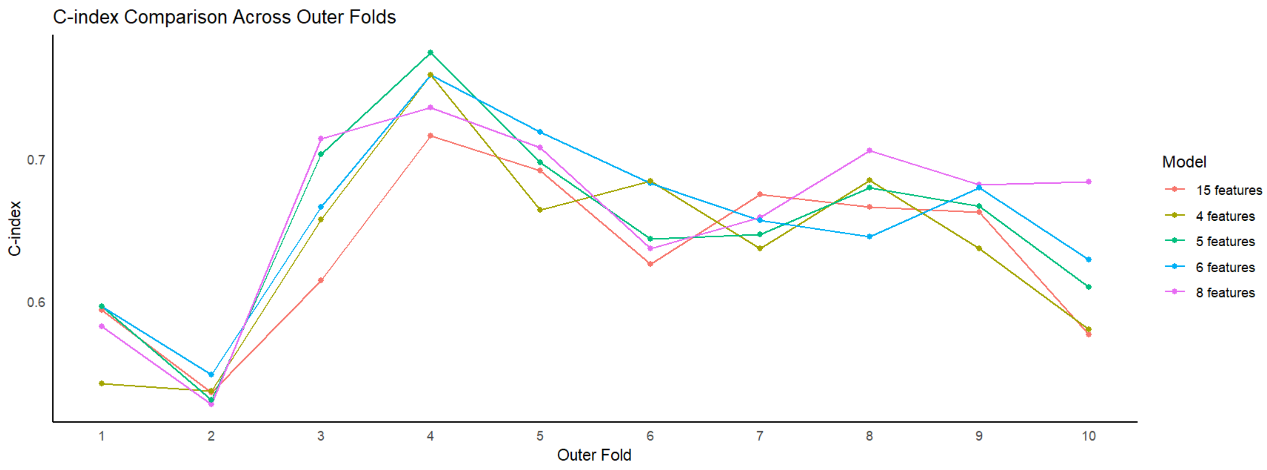

Supplement: Supplementary file 1 [file ijerph-23-00527-s001.zip › ijerph-4195955-supplementary.pdf]
